# Supplementary material for: Effect of NaCl and EDDS on Heavy Metal Accumulation in Kosteletzkya pentacarpos in Polymetallic Polluted Soil
Source: Plants (Basel). 2023 Apr 14;12(8):1656. doi: 10.3390/plants12081656 (PMC10146522; doi:10.3390/plants12081656)
Supplement: Supplementary file 1 [file plants-12-01656-s001.zip › plants-2301283-supplementary.pdf]

Table S1 Mean values of soil characteristics of upper (0-20 cm) soil in the agricultural plot located in Louvain-la-Neuve (Avenue Baudouin I). Each value is the mean of 3 biological replicates.

Parameters	
pH	6.86 ± 0.23
Soil humidity	52 ± 4.8
Electrical conductivity (mS cm^{-1})	0.84 ± 0.1
<u>Element concentration (g kg^{-1} soil)</u>	
Ca	7.1 ± 0.5
Fe	19 ± 0.52
K	18 ± 0.57
Mg	8.4 ± 0.27
Na	7.4 ± 0.16
S	0.37 ± 0.3
